# Integrating Facility-Based Surveillance With Healthcare Utilization Surveys to Estimate Enteric Fever Incidence: Methods and Challenges

**DOI:** 10.1093/infdis/jiy494

**Published:** 2018-09-03

**Authors:** Jason R Andrews, Caitlin Barkume, Alexander T Yu, Samir K Saha, Farah N Qamar, Denise Garrett, Stephen P Luby

**Affiliations:** 1 Division of Infectious Diseases and Geographic Medicine, Stanford University, California; 2 Typhoid Programs, Sabin Vaccine Institute, Washington, District of Columbia; 3 Child Health Research Foundation, Dhaka Shishu Hospital, Dhaka, Bangladesh; 4 Department of Paediatrics and Child Health, Aga Khan University, Karachi, Pakistan

**Keywords:** typhoid, enteric fever, surveillance, incidence, methods, bias

## Abstract

Cohort studies and facility-based sentinel surveillance are common approaches to characterizing infectious disease burden, but present trade-offs; cohort studies are resource-intensive and may alter disease natural history, while sentinel surveillance underestimates incidence in the population. Hybrid surveillance, whereby facility-based surveillance is paired with a community-based healthcare utilization assessment, represents an alternative approach to generating population-based disease incidence estimates with moderate resource investments. Here, we discuss this method in the context of the Surveillance for Enteric Fever in Asia Project (SEAP) study. We describe how data are collected and utilized to adjust enteric fever incidence for blood culture sensitivity, facility-based enrollment, and healthcare seeking, incorporating uncertainty in these parameters in the uncertainty around incidence estimates. We illustrate how selection of surveillance sites and their coverage may influence precision and bias, and we identify approaches in the study design and analysis to minimize and control for these biases. Rigorously designed hybrid surveillance systems can be an efficient approach to generating population-based incidence estimates for infectious diseases.

Enteric fever, an invasive infection with *Salmonella* Typhi or Paratyphi, is estimated to be the cause of more than 15 million illnesses and 150000 deaths annually, the vast majority of which occur in low- and middle-income countries [1–3]. The World Health Organization recently prequalified a new typhoid conjugate vaccine (TCV) and issued a recommendation that TCVs be utilized in the prevention of typhoid and to be prioritized in countries with the highest burden of disease [4]. However, the majority of countries where typhoid is believed to be endemic lack recent and geographically representative data on typhoid incidence. This may represent an obstacle to introducing typhoid vaccines, including decision making concerning which countries or subnational regions to prioritize, as well as assessment of their impact on disease burden.

The most rigorous form of infectious disease burden estimation is a prospective, community-based cohort study. This form of study, commonly used for vaccine trials, involves recruitment of healthy participants in the community who are followed closely for the development of incident typhoid fever. Because individuals with typhoid often seek medical care within days of symptom onset, studies often involve contacting each participant 2–3 times weekly to assess for the development of fever, followed by diagnostic testing for those who report fever. As the incidence of typhoid is typically <1% per year, typhoid cohort studies typically involve enrolling tens of thousands of participants, which is costly and requires substantial research infrastructure [5–7]. Consequently, this approach is relegated largely to short-term research studies and is not a sustainable approach to disease surveillance. Additionally, by actively contacting participants, diagnoses are typically made early in the disease course, prompting therapy that averts severe sequelae. This may alter the spectrum of disease toward more mild cases, which may not be representative of the severity of illness that may be seen in the absence of surveillance. For these reasons, cohort studies may be less attractive for assessing vaccine effectiveness.

A more common surveillance approach is ascertainment of cases presenting to healthcare facilities. This can be done retrospectively or prospectively, assessing culture positivity among patients meeting a suspected case definition. This approach requires fewer resources and, unlike cohort studies, may capture more severe cases as they are more likely to seek care. The major drawback to this method is that it leads to substantial underestimation of the population burden of disease, as only a fraction of all typhoid patients seek care at hospital surveillance sites. Indeed, the majority of individuals with acute febrile illness in typhoid-endemic countries seek care primarily at pharmacies and medical shops, where blood culture capacity is usually not available [8, 9].

There is therefore a gap between cohort studies, which achieve direct population-level estimates of generally mild infections and are resource intensive, and facility-based surveillance, which is inexpensive and underestimates incidence but captures the more severe outcomes that are likely to motivate interventions. A more recently developed approach between these extremes involves pairing facility-based surveillance with population-based healthcare utilization surveys, which we refer to as “hybrid surveillance.” Hybrid surveillance, which has been employed for a number of infectious diseases including typhoid, enables the generation of population-based incidence estimates with considerably fewer resource requirements than cohort studies [10–12]. We have utilized hybrid surveillance in phase 2 of the Surveillance of Enteric Fever in Asia Project (SEAP), an ongoing study that aims to estimate typhoid incidence, clinical spectrum, outcomes, and costs in Bangladesh, Pakistan, and Nepal.

While previous studies and a review have provided an overview of the methods for hybrid surveillance data collection and analysis [10, 11, 13], they have not addressed how to incorporate uncertainty of various components or provided detailed considerations for the design of reliable surveillance systems. In this article, we describe methods used for hybrid surveillance, including considerations concerning surveillance site selection, sample size, and statistical approaches to incorporating uncertainty in various components of this model. We highlight key assumptions underlying this approach and discuss difficulties and potential for biases, as well as new methods for bias correction. The methods described here were developed in the context of enteric fever surveillance, but may have broader applicability to surveillance for other diseases.

## HYBRID SURVEILLANCE FRAMEWORK AND COMPONENTS

**Figure 1.**
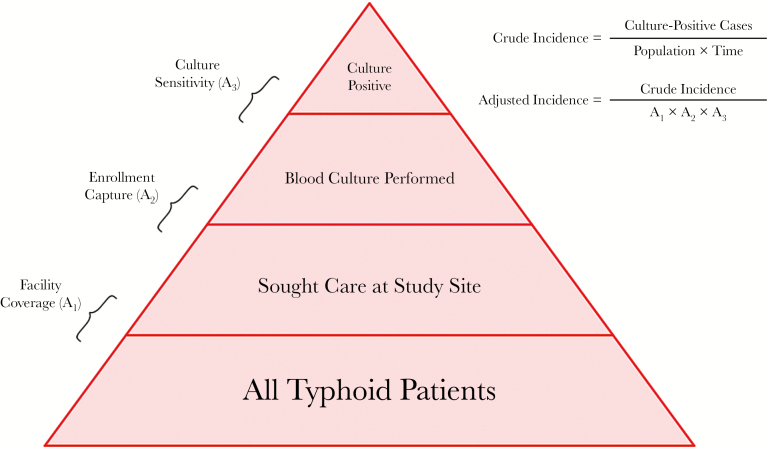
Typhoid disease pyramid, where the base represents all typhoid patients in a catchment area and the apex represents culture-positive cases detected at study sites. Culture sensitivity is estimated from the literature, enrollment capture is estimated at facilities, and facility coverage is estimated by a household survey. These factors are utilized to adjust the crude incidence.

Hybrid surveillance may be thought of as characterizing the layers of a disease detection pyramid, where all typhoid patients within a catchment area represent the base, and culture-confirmed cases detected at surveillance facilities represent the apex (Figure 1). A fraction of all typhoid patients in the community will seek care at study sites, a proportion of those are recruited and enrolled into the surveillance system, and the sensitivity of blood culture will determine how many cases are detected among those enrolled. Hybrid surveillance involves adjusting cases detected for these factors. Here, we describe the overall surveillance approach, including how each of these parameters is estimated, and then describe how these estimates are utilized to generate incidence estimates.

First, the overall study population must be identified, usually in a geographically circumscribed area. It is possible to first identify an area of interest, and then choose commonly utilized healthcare facilities within that area to establish surveillance, but often the surveillance sites with the capacity for microbiological diagnoses are identified first and the catchment area is subsequently characterized. The catchment area of a health facility generally depends on the disease of interest, with more severe conditions having a larger catchment area for a well-resourced facility; as a result, the catchment should area be defined with respect to the disease that is being studied. Importantly, surveillance catchment areas are not drawn to identify the entire geographic expanse of patients’ homes who utilize these facilities. Rather, catchments for surveillance are drawn by the surveillance team to optimize measurement of incidence. For the SEAP phase 2 studies, the catchment areas were constructed by mapping either suspected or culture-confirmed typhoid cases from selected sentinel surveillance sites in recent years. After mapping cases, geographical boundaries are identified that contain the majority of cases and represent well-recognized administrative boundaries (eg, city ward), enabling patients presenting to facilities to easily identify whether they live within the study catchment.

After the catchment area has been specified, healthcare utilization in that population is assessed by means of a household survey. This is often done concomitantly with facility-based surveillance. In the SEAP study, a multistage, cluster design was utilized to select a representative subset of all households in the catchment area for survey. The questionnaire focuses on whether any individuals in the household met a simple, standardized definition for suspected enteric fever (fever ≥3 days) within a time period in which recall is expected to be reliable (past 8 weeks). As recall regarding hospitalization is thought to be reliable for a longer recall period, additional questions are asked about hospitalization within the past year for febrile illness. Questions concerning severity of the febrile illness, demographics, household assets, and water source are asked to enable adjustments for differences in populations who seek care at the study site and those who do not, as described below. This survey may also be used to estimate the population size and age structure for the catchment area, if recent census data corresponding to that area are not available.

Additionally and typically concomitantly, facility-based surveillance for enteric fever is established at one or more sentinel sites within the catchment area. Patients seeking care at sentinel sites who meet study inclusion criteria are recruited and offered enrollment into the study. For the SEAP study, enrollment criteria are as follows: an individual, residing within the catchment area, who presents to the outpatient or emergency department with fever for ≥3 consecutive days within the past week. A blood culture is offered to all enrolled individuals, and confirmed enteric cases are defined as individuals with a blood culture positive for typhoidal *Salmonella*. Even under study conditions, not all individuals presenting to sentinel sites who meet the enrollment criteria will be enrolled, due to missed cases (eg, when research staff are not present), difficulties with collecting blood, or declined consent. Logs are utilized to record the numbers of eligible patients who were not enrolled, to enable adjustment for this missing proportion. Systematic random sampling (eg, enrolling every fifth eligible patient, or enrolling every other day) may also be performed if resources are constrained, though care must be taken to ensure that sampling remains systematic and selection biases are not introduced.

The final adjustment factor accounts for the sensitivity of the diagnostic; for enteric fever, blood culture remains the primary reference standard due to nearly perfect specificity and the lack of highly specific alternative diagnostics [14]. Estimates for the sensitivity of blood culture are derived from published literature, predominantly studies in which bone marrow culture (a highly sensitive and specific approach, but unsuitable for large-scale surveillance due to its invasive nature) was performed. A recent meta-analysis reported a composite estimate of 61% (95% confidence interval [CI], 52%–70%) [15]. Multiple factors influence the sensitivity of blood culture including the duration of fever prior to collection, recent antibiotic use, blood volume collected, and the culture media, including use of antibiotic binding resins. Ongoing work is aimed at characterizing the influence of these parameters, which could enable better estimates for culture sensitivity according to these factors at each study site.

## ESTIMATING INCIDENCE FROM HYBRID SURVEILLANCE

Crude incidence estimates from facility-based surveillance are derived by dividing the total number of culture-confirmed enteric fever cases by the product of the population and time:

$$\text{Crude Incidence}=\frac{\text{Cases}}{\text{Population}\times \text{Time}}$$

Adjusted incidence can be estimated by dividing the crude incidence by the 3 adjustment factors:

$$\text{Adjusted Incidence}=\frac{\text{Crude Incidence}}{{\text{A}}_{1}\times {\text{A}}_{2}\times {\text{A}}_{3}}$$

Here, A_1_ is the proportion of individuals in the community meeting the case definition who seek care at the study site (“facility coverage”), A_2_ is the proportion of patients presenting to the facility and meeting the case definition who are enrolled (“enrollment capture”), and A_3_ is the sensitivity of culture. In some situations, partially adjusted incidence estimates, in which not all factors are adjusted for, may be of interest. For example, if there is concern about biases in healthcare utilization, estimates that adjust only for culture sensitivity and facility-based enrollment may be desired, to provide a minimum estimate for disease incidence. Estimates are often presented with stratification by age, using age-specific case counts, catchment population, and adjustors.

To characterize uncertainty in incidence while reflecting the uncertainty in each of these adjustments, we perform Monte Carlo sampling from distributions of cases (binomial) and adjustment factors (beta). When population size is estimated directly from the healthcare utilization survey, rather than census estimates, uncertainty in population size may be incorporated into the model.

## FACILITY COVERAGE AND SAMPLE SIZE

**Figure 2.**
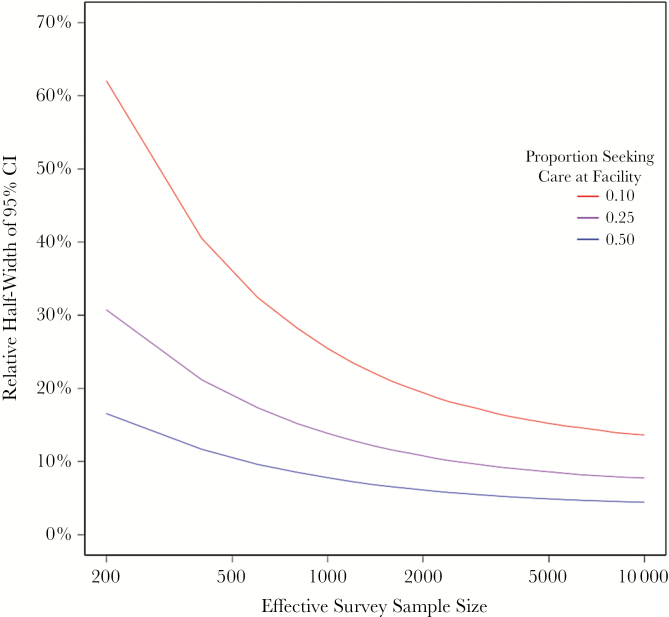
Relative half-width of the 95% confidence interval (CI) achieved by hybrid surveillance as a function of effect sample size (ie, number of households with an individual meeting study definition for febrile illness) and fraction of patients captured at study sites. Here, incidence is 500 per 100000 and the population size is 500000. A higher proportion of participants seeking care at the facility improves the precision of the adjustment factor estimate as well as the precision in the numerator, as more typhoid cases present to the facility. A larger healthcare utilization sample size can improve precision in the adjustment factor estimate, but greater uncertainty in the incidence estimate remains due to uncertainty in the case number.

The proportion of patients with enteric fever who seek care at the surveillance sites (“facility coverage”) is a critical determinant of uncertainty in incidence estimates. Smaller facility coverage fractions require substantially larger healthcare utilization surveys to achieve equivalent precision in incidence estimates. To illustrate this, we simulate a hypothetical community with a population of 500000 and an annual enteric fever incidence of 2500 cases (500 per 100000). We vary the proportion that present to surveillance sites (10%, 25%, 50%) and assume that care seeking is binomially distributed. We compare precision of incidence estimates according to effective sample size (households with a participant meeting the fever definition in past 8 weeks and cluster adjusted sample) for each of these 3 facility-based capture rates (Figure 2), ignoring uncertainty introduced by culture sensitivity at this time.

For communities in which 50% of individuals with suspected enteric fever seek care at the study sites, relative precision of ±10% (95% CI, 450–550 per 100000) can be achieved by an effective sample size of 550 households. By contrast, communities in which 25% and 10% of individuals seek care at study sites would have errors of ±27% and 34%, respectively, at that same sample size. Achieving ±10% error would require an effect sample size of 2500 for communities with 25% seeking care at study sites; this ±10% error rate would not be possible for communities with 10% seeking care at study site. The error does not approach 0 because of the remaining uncertainty in the numerator. For an equivalent population size, a lower proportion of individuals seeking care at the study site results in fewer enrolled cases and a larger degree of uncertainty around crude rates. In other words, lower facility coverage results in greater uncertainty in both the numerator (cases, which are fewer when this proportion is low) and the multiplier. A larger healthcare utilization survey can offset the latter, but a longer study (to enroll more cases) is required to offset the former.

## ADDRESSING POTENTIAL BIASES

The hybrid surveillance pyramid has all typhoid patients as the base, and the second level are those that seek care at the study sites. The key multiplier here is the proportion of all typhoid patients who seek care at the study sites; however, what is measured by the healthcare utilization survey is the proportion of individuals meeting a typhoid suspect case definition (fever ≥3 days) who seek care at the study site. A fundamental assumption of this method has been that care-seeking patterns among typhoid suspects provide an unbiased estimate for care seeking among typhoid patients. If individuals with typhoid are more (or less) likely to seek medical care, or to seek care at study sites, than individuals with other febrile illnesses lasting 3 days, this estimate may be biased. Bias in the estimate of this parameter in turn leads to biased estimates of enteric fever incidence; specifically, if typhoid patients are more likely to seek care at study sites than patients with other causes of fever, typhoid incidence would be overestimated. The converse would also be true.

We hypothesized that individuals with typhoid may have more severe symptoms than those with other febrile illnesses (eg, viral infections) and therefore may be more likely to present to tertiary facilities where surveillance is often conducted. In the SEAP study, we therefore introduced two questions aimed at assessing the severity of illness among individuals meeting the suspected typhoid definition. First, we inquired about how many hours the individual spent in bed on their worst day of illness. Second, we inquired about how many days the individual was unable to conduct their usual activities due to illness. We asked these questions in both the healthcare utilization survey, as well as among culture-confirmed patients and culture-negative patients at the surveillance site. Additionally, we hypothesized that there may be associations between household wealth and typhoid risk, as well as between wealth and healthcare seeking. For bias to occur, variables that are associated with typhoid must also be associated with care seeking, and these associations must be conditionally independent.

**Figure 3.**
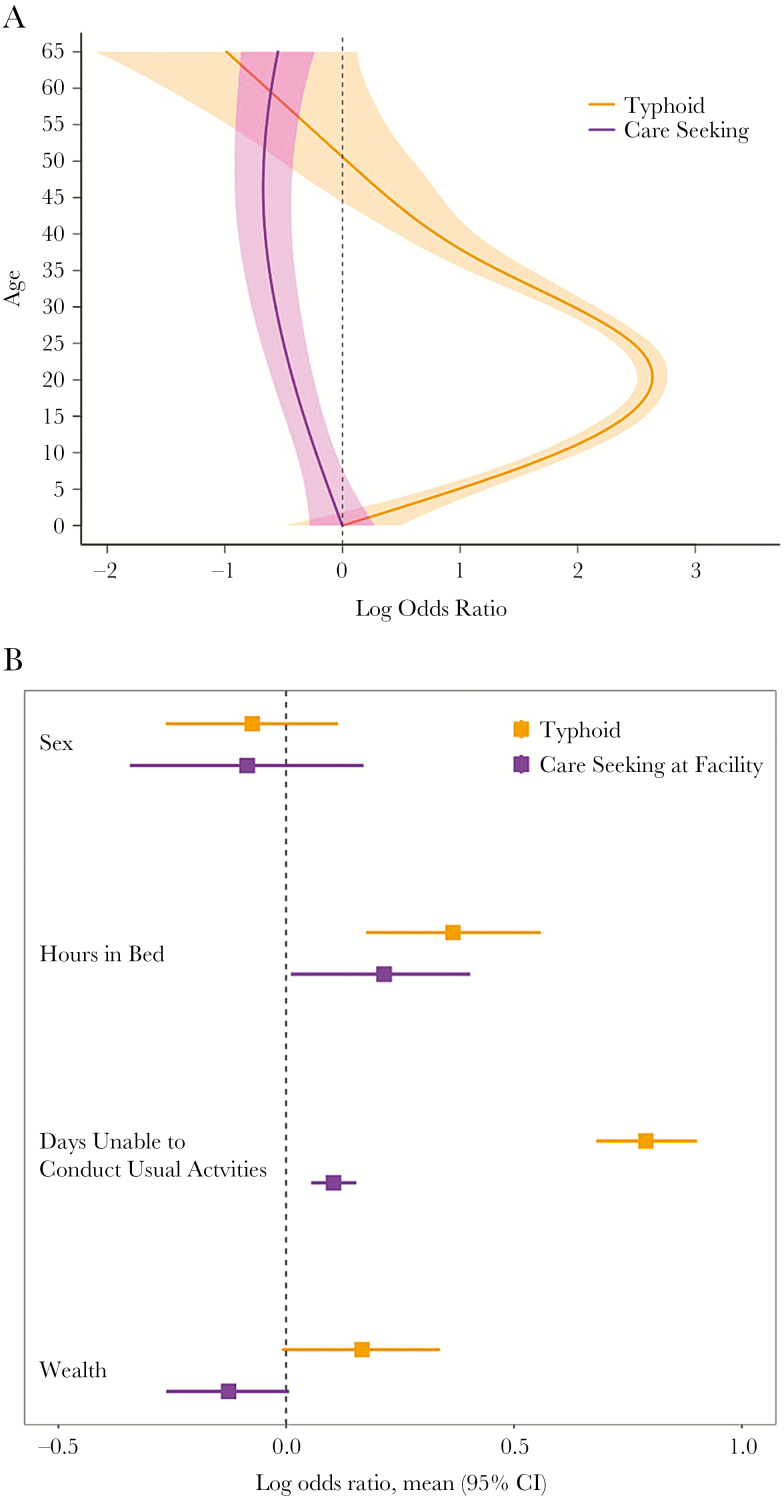
*A*, Log odds ratio for typhoid and care seeking, by age, in Surveillance for Enteric Fever in Asia Project (SEAP) data from Nepal. The reference group is age 0. *B*, Log odds of typhoid and care seeking by demographic and severity indicators in SEAP data from Nepal. Abbreviation: CI, confidence interval.

Here, we present preliminary data from Nepal to illustrate this process of assessing for biases. First, we compared the odds of seeking care and the odds of having culture-confirmed typhoid, according to age, using generalized additive models. We found strong and differential associations, which were nonlinear (Figure 3A). These findings clearly support the need for age-stratified analyses to minimize biases with this method. We found that both severity questions correlated positively with both typhoid and care seeking at study site, indicating potential bias in the estimate of this factor that would cause *overestimation* of incidence (Figure 3B). Conversely, we found that wealthier individuals were more likely to have typhoid, but less likely to seek care at study sites, a potential bias that would lead to *underestimation* of incidence.

To adjust for these potential biases, we perform a 2-stage regression model, in which we first perform multivariable logistic regression to model the relationship between demographic and severity factors and typhoid culture positivity using facility-based data. We then use this model to estimate typhoid culture positivity among febrile individuals in the catchment area by utilizing the distribution of demographic and severity data from the healthcare utilization survey. Dividing the observed odds of typhoid from the facility data with the predicted odds of typhoid in the healthcare utilization survey results in a summary odds ratio (OR) for typhoid among care seekers compared with the general population. This OR can then be applied to the adjusted incidence equation above to correct for differences in care seeking.

In the preliminary Nepal data, we observed no statistically significant differences in predicted typhoid prevalence between those who sought care at study sites and those who did not (*P* = .13), due to the offsetting effects of illness severity and wealth. However, it should be noted that the absence of differences does not indicate that there are no differences, but rather that the specified model did not identify them, which precludes their adjustment in incidence estimates.

**Figure 4.**
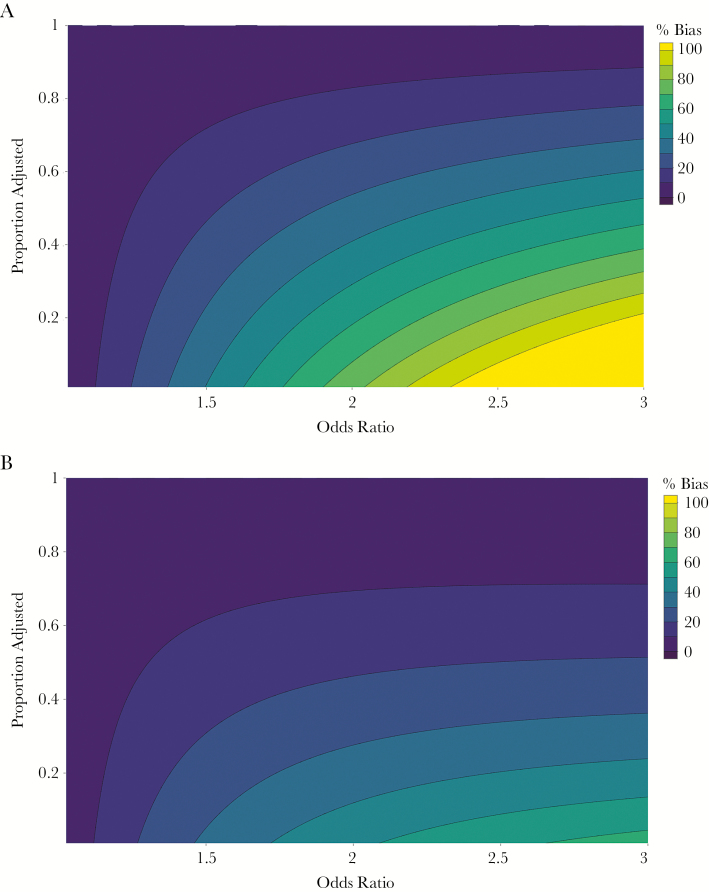
Percentage bias in incidence estimate as a function of the odds ratio for care seeking at study facilities among typhoid patients compared with nontyphoid patients, and the percentage adjusted for in the analysis. The proportion seeking care at the study site is 0.1 (*A*) and 0.5 (*B*).

To illustrate the potential magnitude of this problem more generally, we estimate the bias in incidence estimates as a function of (1) the OR of typhoid among individuals seeking care compared with those not seeking care at study sites; (2) the proportion of the population seeking care at the study sites; and (3) the proportion of care-seeking differences (ie, fraction of OR − 1) that can be identified and adjusted for by the regression method described above (Figure 4). We find that substantial bias (>70%) may be seen when the OR for care seeking is high (>2), the proportion seeking care at study site is low (10%), and the proportion adjusted for is low (10%). By contrast, even with substantial differences in care seeking (OR of 3), if 50% of the difference can be adjusted for in the model and 50% of participants seek care at the study site, the bias in incidence estimates would be around 15%. These findings highlight the importance in establishing surveillance at sites with high coverage for febrile illness in the catchment population and characterizing differences in populations who seek care at study sites and those who do not.

An additional source of bias manifests when individuals, meeting enrollment criteria for suspected typhoid, who are enrolled at study sites differ from those who are not enrolled with respect to typhoid risk. This may occur due to clinicians or study staff preferentially enrolling individuals suspected to have enteric fever or greater patient propensity to consent for blood draw when they have more severe illness. In practice, we have observed clinicians discouraging patient enrollment in patients whom they believe are unlikely to have enteric fever; however, we note that half of culture-confirmed cases occur in patients in whom the primary diagnosis was an alternative infection. At some surveillance sites, clinicians have reported not enrolling patients whom they are confident have enteric fever; the bias therefore may act in either direction. The primary mechanism for addressing this source of bias is through education of clinicians and study staff about these data and encouraging consistent application of suspected typhoid definitions. If enrollment rates are low due to blood culture concerns by patients or clinicians, the potential for bias is greater. In such settings, enrollment of individuals who decline (or whose clinician advises against) blood culture is useful to collect demographic and clinical data along with clinician diagnoses. These data may be compared with data from enrolled participants with culture data and can be used to generate regression-based estimates of typhoid prevalence among individuals who do not have culture performed.

## CONCLUSIONS AND FUTURE DIRECTIONS

Hybrid surveillance represents a lower-cost alternative to cohort studies for generating population-based incidence estimates for enteric fever and other infectious diseases. For this reason, the ongoing SEAP and Severe Enteric Fever in Africa (SETA) studies are utilizing this approach. Here, we illustrated the framework, methods, and statistical considerations for estimating disease incidence and incorporating uncertainty in various components of the surveillance pyramid. We identify several important considerations in hybrid surveillance design, implementation, and data analysis that have implications for the precision and accuracy of estimates.

First, when the sentinel surveillance sites capture a lower proportion of typhoid cases, this results in lower precision around typhoid incidence estimates, which may be only partially offset by a larger healthcare utilization survey or longer study. Second, we highlight a fundamental assumption in this surveillance method, which is that individuals meeting a suspected typhoid definition (fever ≥3 days) who present to surveillance sites are representative of those who do not present to those sites, with respect to their likelihood of having typhoid. In reality, we might anticipate that individuals with typhoid, compared with those with other illnesses, may have differences in illness severity and sociodemographic factors that influence care-seeking patterns. These differences may lead to substantial bias in incidence estimates, and the magnitude of this bias is inversely proportionate to the proportion of patients who seek care at the study site. When a smaller proportion of care-seeking individuals are used to infer risk about a larger proportion of individuals not seeking care at the study site, the impact of small differences is amplified.

We identify 3 potential approaches to minimizing this risk of bias. The first is establishing surveillance sites that cover a higher proportion of typhoid-like illnesses, which can be done by either involving more sites or utilizing a smaller or more carefully constructed catchment area, in which the care-seeking proportion is higher. Achieving higher coverage by sentinel sites should reduce bias and improve precision of estimates. The second is utilizing a more specific case definition for enrollment at study sites and inference in the healthcare utilization survey. For example, hybrid surveillance has been successfully performed for Japanese encephalitis by utilizing a suspected case definition of fever with altered mental status or new-onset seizures, which are relatively uncommon and specific symptoms for meningoencephalitis, and there is less likely to be differential care seeking according to type of encephalitis [13]. A key challenge with typhoid is that the disease produces highly nonspecific manifestations that are variable and difficult to distinguish from other infections [16–18]. A recent study from Nepal demonstrated that even clinician diagnoses have <10% positive predictive value [19]. Attempting to select a more specific case definition for typhoid (eg, ≥4 or 5 days, or absence of focal symptoms) would reduce the number of eligible participants at the surveillance sites and in the healthcare utilization survey, offsetting efficiency gains, and could potentially introduce additional biases into the study design by focusing on a narrow set of disease manifestations. The most tenable approach to addressing potential biases in care seeking may be in collecting data to identify sociodemographic and severity factors that predict typhoid risk and care seeking, which may enable adjustment as outlined above.

Biases may also be introduced by selective enrollment at the sentinel surveillance sites, if individuals with typhoid are more likely to self-select for consent or be recruited by clinicians or study staff. While it may be possible to adjust for these biases by characterizing eligible nonenrolled patients, the best approach is to educate clinicians and study staff about the poor predictive value of empirical diagnoses, to maximize recruitment rates. If recruitment remains low, the potential for bias is high, and obtaining clinical data on eligible individuals who are not cultured may be useful for comparison with that of individuals who are cultured.

**Figure 5.**
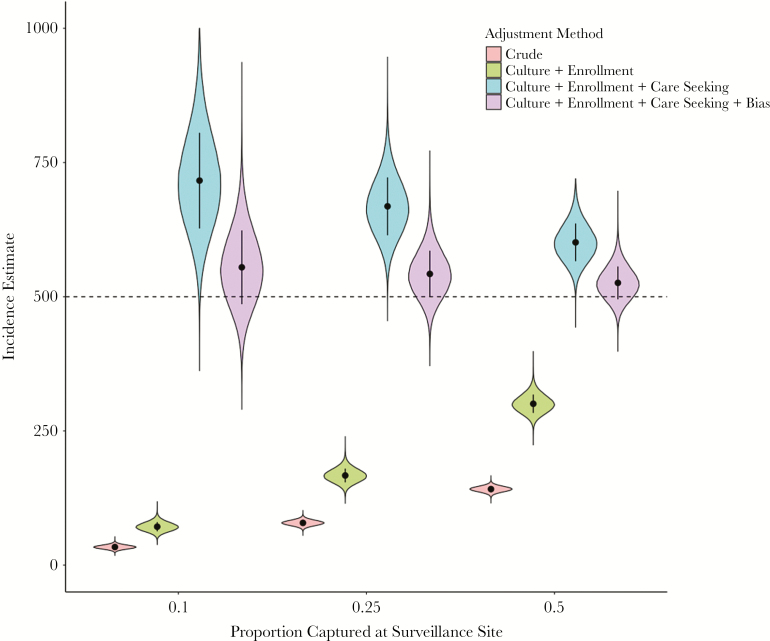
Incidence estimates and uncertainty intervals according to adjustment method and proportion captured at the surveillance site. We assume 1 year of surveillance in a population of 100000 individuals with typhoid incidence of 500 per 100000, a healthcare utilization assessment of 5000 households, and odds ratio of care seeking at study sites for typhoid patients (bias) of 1.5. Culture sensitivity (assumed 59% [95% confidence interval, 54%–64%]); proportion of eligible individuals at study site enrolled (assumed 80%); adjustment for care seeking (varied, x-axis); bias: adjustment for biases in healthcare seeking (correction of 67% of bias). The dashed line represents the true prevalence.

Due to the potential for biases in various components of hybrid surveillance, it may be advisable to present multiple estimates for disease incidence, highlighting the influence of various assumptions (Figure 5). These include crude, culture-confirmed incidence, incidence adjusted for culture sensitivity and facility-based enrollment, incidence further adjusted for healthcare utilization, and, finally, incidence adjusted for all of these factors along with bias correction for differences in care seeking. We note that further adjustments may be possible and desirable, if the requisite data are available. For example, blood volume utilized for culture and antibiotic use prior to blood culture likely influence culture sensitivity; if this relationship is well characterized and these data are available in the surveillance system, they may be used to further improve accuracy of incidence estimates.

While case-based surveillance remains the only valid source of data for typhoid burden assessment at present, we note that seroepidemiologic approaches are under development. Most of the experience to date has been with measuring immunoglobulin G to Vi antigen [20–22], which has been imperfect and may increasingly be undermined as a marker of disease burden due to the rollout of Vi-conjugate vaccines. Alternative serologic markers have demonstrated promise for identifying acute enteric fever and are being assessed for a potential role in seroepidemiology [23, 24].

Hybrid surveillance is not without limitations, but for relatively uncommon diseases (<1% per year), it represents a powerful and efficient means for generating estimates of disease incidence at a fraction of the cost of cohort studies. With periodic healthcare utilization assessments alongside continuous hospital surveillance, longitudinal monitoring of trends in incidence of enteric fever may be maintained in resource-limited settings, providing policy-relevant data, including in the context of vaccine introduction. Careful attention to potential sources of bias in system design, implementation, and data analysis can improve the accuracy of incidence estimates and result in rigorous and sustainable systems for population-based infectious disease surveillance.

## Notes

***Supplement sponsorship.*** This article is part of the supplement “Surveillance for Enteric Fever in Asia Project,” sponsored by the Sabin Vaccine Institute.

***Potential conflicts of interest.*** J. R. A. and S. P. L. have received grants from the Bill & Melinda Gates Foundation. All other authors report no potential conflicts of interest. All authors have submitted the ICMJE Form for Disclosure of Potential Conflicts of Interest. Conflicts that the editors consider relevant to the content of the manuscript have been disclosed.
